# Feasibility and advantages analyses of wedge resection without mesentery detached approach applied to closure of loop ileostomy

**DOI:** 10.1186/s12893-022-01661-5

**Published:** 2022-06-02

**Authors:** Hai-Quan Qin, Jian-Kun Liao, Wen-Tao Wang, Ling-Hou Meng, Zi-Gao Huang, Xian-Wei Mo

**Affiliations:** 1 Division of Colorectal and Anal, Department of Gastrointestinal Surgery, Guangxi Medical University Cancer Hospital, No.71, Hedi Road, Qingxiu District, Nanning, 530021 Guangxi Autonomous Region China; 2 Guangxi Clinical Research Center for Colorectal Cancer, Guangxi Medical University Cancer Hospital, No.71, Hedi Road, Qingxiu District, Nanning, 530021 Guangxi Autonomous Region China

**Keywords:** Closure of loop ileostomy, Diagnosis-related groups, Low rectal cancer, Wedge resection, Transverse suture

## Abstract

**Objectives:**

To evaluate the feasibility and advantages of wedge resection plus transverse suture without mesentery detached approach applied to loop ileostomy closure by analyzing the surgical data and the incidence of postoperative complications of patients undergoing this procedure.

**Methods:**

We performed a retrospective analysis of the hospitalization data of patients who underwent ileostomy closure surgery and met the research standards from January 2017 to April 2021 in Guangxi Medical University Cancer Hospital; all surgeries were performed by the same surgeon. The perioperative data were statistically analyzed by grouping.

**Results:**

In total, 65 patients were enrolled in this study, with 12 in the wedge resection group, 35 in the stapler group, and 18 in the hand suture group. There was no significant difference in operation time between the wedge resection group and stapler group (P > 0.05), but both groups had shorter operation time than that in the hand suture group (P < 0.05). The postoperative exhaustion time of wedge resection group was earlier than that of the others, and cost of surgical consumables in the wedge resection group was significantly lower than that in the stapler group, all with statistically significant differences (P < 0.05). By contrast, there were no statistically significant differences in postoperative complication incidences among the three groups.

**Conclusions:**

The wedge resection plus transverse suture without mesentery detached approach is safe and easy for closure of loop ileostomy in selected patients, and the intestinal motility recovers rapidly postoperatively. It costs less surgical consumables, and is particularly suitable for the currently implemented Diagnosis-Related Groups payment method.

## Introduction

With the development of treatment technologies such as minimally invasive laparoscopy and internal rectal sphincterotomy, the indications for anus-preserving surgery in patients with rectal cancer have been expanded, and the anus preservation rate for patients with low rectal cancer has been greatly improved [1–3]; however, it also increases the risk of postoperative anastomotic leakage [4, 5]. Once anastomotic leakage occurs, it will lead to serious complications and consequences, such as pelvic infection, anastomotic stenosis, and defecation dysfunction [6]. To reduce the incidence and the associated serious consequences, the establishment of temporary loop ileostomy to shunt stool is a common preventive measure [7, 8]. Under normal circumstances, 3 months after radical operation and full rectal anastomotic stromal healing, the ileostomy closure surgery is performed to close the temporary stoma and restore normal anatomy and physiology. In the past, the commonly used surgical approaches for ileostomy closure surgery included the hand suture approach (end-to-end anastomosis) and the stapler approach (end-to-end, end-to-side, or side-to-side anastomosis), each with their own advantages and disadvantages [9–13]. Although many reports have compared and analyzed the advantages of the above approaches, the first-choice surgical treatment has yet to be made clear. Particularly, the stapler anastomosis approach is widely welcomed owing to its simple operation, shorter operation time, and firm anastomosis; however, its application of expensive staplers leads to high anastomotic costs, which further increases the patients’ total hospitalization costs and the country’s medical insurance costs [9, 14].

At present, medical insurance expenditures impose a great burden in the financial expenditures of countries worldwide, especially in developing countries with a large population base such as China, wherein medical insurance expenditures are large and the country’s financial burden is heavy [15]. Therefore, to reduce the national financial burden, China has begun to implement the diagnosis-related groups (DRG) payment method to reduce the average hospitalization expenses of patients.

Generally, the wedge resection (on the opposite side of the mesentery) plus transverse suture approach has been used in intestinal fistula repairing, with better intestinal conditions and less soiling [16]. Despite this, in previous ileostomy closure surgeries, we have found that in cases where intestinal adhesion with local skin and mucosal inflammation are mild, the adhesion between the intestine and the abdominal wall can be decreased, while avoiding intestinal injury of the stoma. Additionally, the ileostomy ring resembles a fistula with no evident infection and with good local intestinal tract conditions, making it feasible for local excision and repair. From an economic perspective, if the wedge resection plus transverse suture without mesentery detached approach can be applied to ileostomy reversal, it will significantly reduce the patients’ hospitalization costs and the national medical insurance burden. However, whether the wedge resection plus transverse suture without mesentery detached approach is safe and feasible for ileostomy closure has been rarely reported.

Therefore, this research applied the wedge resection plus transverse suture without mesentery detached approach to ileostomy reversal and compared it with the two surgical approaches—the stapler side-to-side anastomosis and hand suture end-to-end anastomosis—in terms of operation time, intraoperative blood loss, postoperative exhaustion time, postoperative complications within 30 days, cost of surgical consumables, and other observation indicators, to analyze the feasibility, safety, and economic benefits, among others of this approach.

## Materials and methods

This study had a retrospective design conducted in the Department of Gastrointestinal Surgery, Guangxi Medical University Cancer Hospital, wherein the hospitalization data were collected from patients who underwent ileostomy closure surgery performed by the same attending physician from January 2017 to April 2021. Patients with other serious gastrointestinal diseases, severe cardiovascular and cerebrovascular diseases other than the underlying disease, or those with missing hospitalization data were excluded from this study. According to the different surgical approaches of ileostomy reversal, the patients were divided into the wedge resection (transverse suture) group, stapler (side-to-side anastomosis) group, and hand suture (end-to-end anastomosis) group.

All patients were informed and signed surgical consent before surgery. All patients underwent the same perioperative treatments except for the different surgical approaches. There were no abdominal drainage tubes or subcutaneous drainage strips indwelling during operation. After surgery, all patients underwent fasting, wherein liquid diet could be taken after exhausting through anus, and they received symptomatic treatments, such as nutritional support, antibiotics, wound dressing, etc.

### Stapler and hand suture approaches

The stoma was temporarily closed by continuous or purse-string sutures. A fusiform incision was made around the stoma and the stoma was separated gradually from the abdominal wall until the intestinal segment of the stoma could be dragged out of the abdominal cavity. Stapler and hand suture approaches both required wiping out the intestines and ligating corresponding mesostenium of stoma segment, resulting in a certain distance between the anastomotic stoma and the mesostenium margin. This distance should not be greater than 1.0 cm to avoid anastomotic leakage caused by anastomotic ischemia.

Then in the stapler approach, 2 pieces of 80 mm anastomosis nails were required, one for performing side-to-side anastomosis, and the other for closure of the common opening end.

And in the hand suture approach, which is a traditional standard way of anastomosis in our department, end-to-end anastomosis was performed manually with intermittent full layer suture by absorbable sutures.

### Wedge resection plus transverse suture without mesentery detached approach

**Fig. 1:**
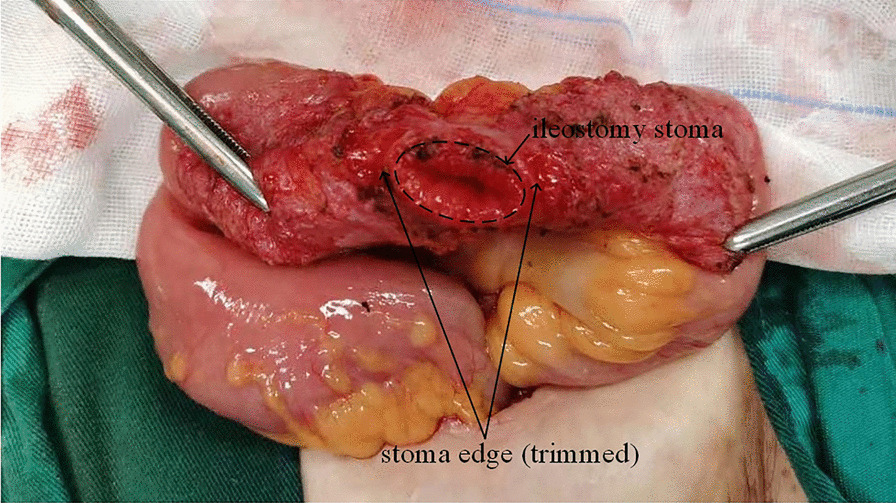
The picture shows that intestinal segment of the stoma was fully freed, afterwards, the stoma edge was trimmed, and the skin and tissue were removed

**Fig. 2:**
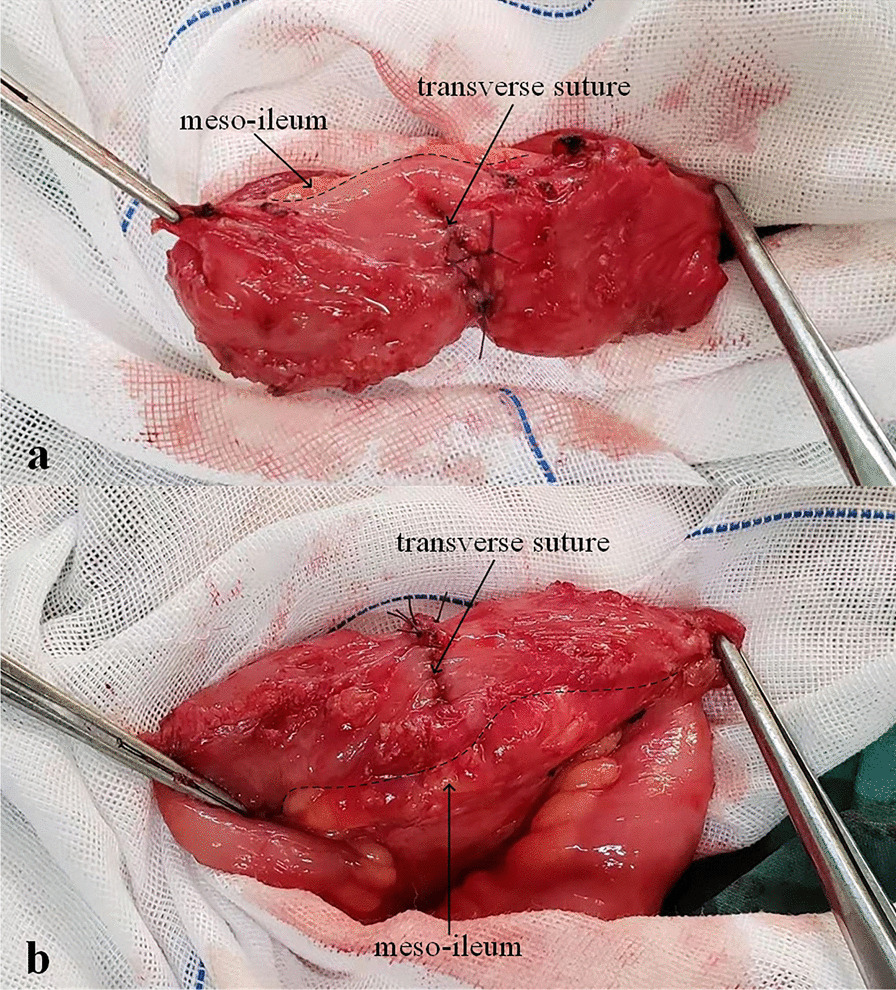
Intermittent full-thickness sutures were performed along the transverse axis of the intestinal tube. **a** Front view. **b** Side view

The main technical points were as follows: The stoma was temporarily closed by continuous or purse-string sutures by sewing the skin without damage to the intestinal wall, and this is one of the keys. Then a fusiform incision was made around the stoma and the intestinal segment of the stoma was then fully freed; afterwards, the stoma edge was trimmed in order to remove the adhesive skin and tissues and keep a healthy intestinal wall (Fig. 1). After resection, intermittent full-thickness sutures were performed along the transverse axis of the intestine (Fig. 2a, b).

**Fig. 3:**
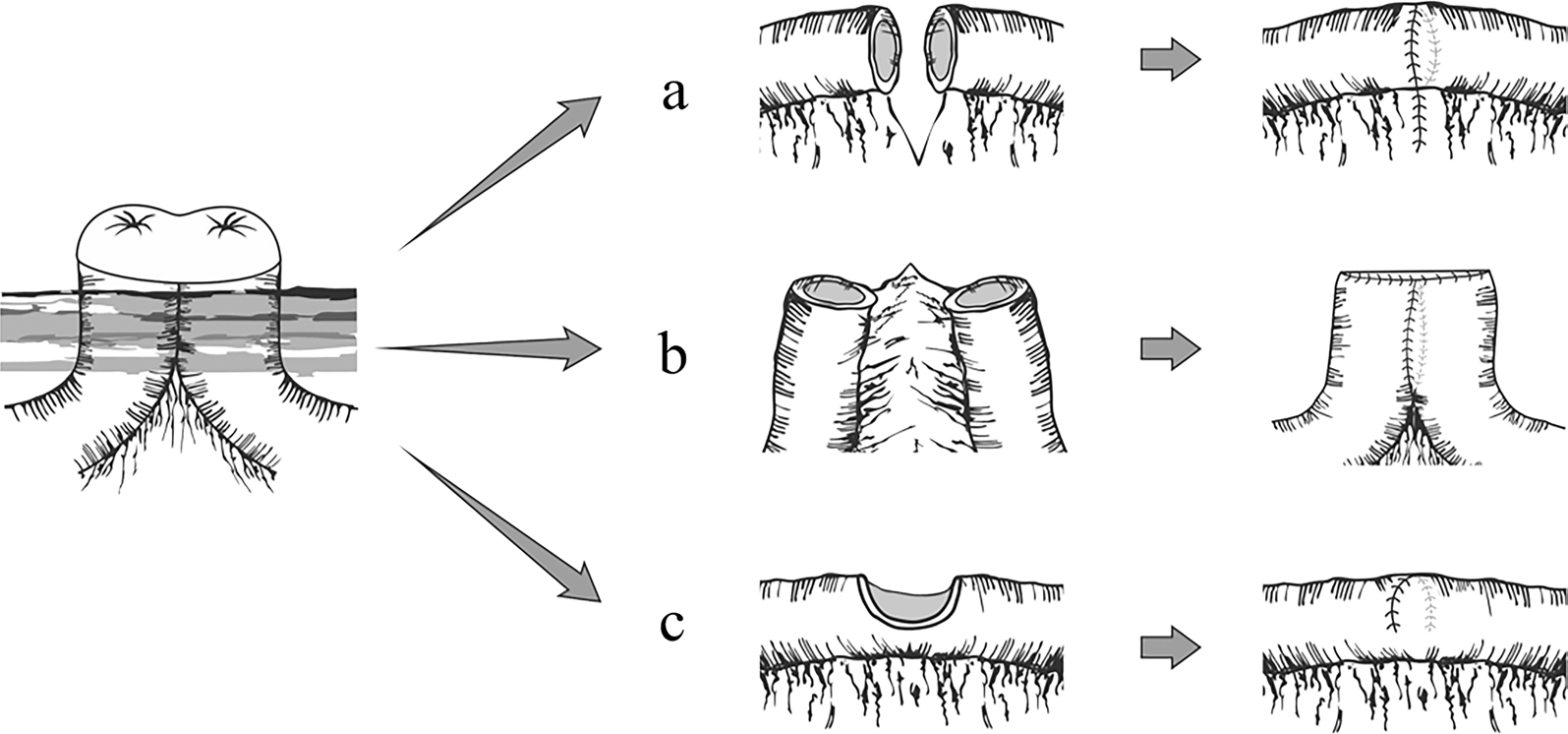
The picture shows the schematic diagram of three surgical approaches in this paper. **a** Hand suture (end-to-end anastomosis) approach. **b** Stapler (side-to-side anastomosis) approach. **c** Wedge resection plus transverse suture without mesentery detached approach

The sarcoplasmic layer was sutured intermittently to reinforce the anastomotic stoma in all three groups routinely. And schematic diagram of three surgical approaches is shown in Fig. 3.

### Preliminary criteria for patient selection of wedge resection

(1) There was no obvious edema in the intestinal segment of stoma; (2) No ostomy related complications such as necrosis and surrounding infection occurred during the existence of the stoma; (3) Preoperative examination showed no formation of parastomal hernia; (4) During operation, the ostomy was separated smoothly without obvious intestinal damage, in particular, full-thickness injury to the intestinal wall; (5) There was no radiation enteritis in patients with neoadjuvant radiotherapy before radical surgery which resulting in intestinal hyperemia, edema, thickening and erosion etc. (6) There was no significant difference in the diameter of intestinal lumen at both ends of the anastomosis.

If the above criteria were met, wedge resection plus transverse suture without mesentery detached approach would be adopted by the surgeon; otherwise, stapler approach or hand suture approach should be chosen according to the surgeon's judgment.

### Observation indicators

The hospitalization data of the included patients were collected, with the main observation indicators, namely, operation time, intraoperative blood loss, surgical consumables cost, postoperative exhaustion time, postoperative length of hospital stay, secondary operation rate, readmission rate, and postoperative complication incidence, especially the occurrence of anastomotic bleeding, anastomotic leakage, continuous vomiting, abdominal distension, and intestinal obstruction within 30 days after surgery. Particularly, the second operation rate and readmission rate were defined as the proportion of reoperation and readmission for the required treatment due to complications after ileostomy closure surgery within 30 days, respectively. And postoperative exhaustion time was defined as the time of first exhaust or defecation after surgery.

### Statistical analysis

SPSS 25.0 statistical software (IBM Corp, Armonk, NY, USA) was used in this study. The measurement data were expressed as (x ± s) or median and range. One-way ANOVA was used for comparing among the groups of measurement data. The enumeration data were expressed as percentages (%), and Pearson Chi-square test or Fisher’s exact test was used as the statistical method for them, and comparison among groups was analyzed by Bonferroni test. P < 0.05 indicated that the differences in all the tests were statistically significant.

## Results

In total, 65 patients were included in this study. According to the different surgical approaches, 12 were included in the wedge resection group, 35 were in the stapler group, and 18 were in the hand suture group. The baseline data of the three groups of patients, namely, gender, age, body mass index (BMI), underlying diseases, and chemoradiotherapy history are shown in Table 1.

**Table 1:** Basic baseline characteristics in the three groups

	Wedge resection (n = 12, %)	Stapler (n = 35, %)	Hand suture (n = 18, %)	*P* value
Gender				0.454
Male	7 (58.3)	27 (77.1)	13 (72.2)	
Female	5 (41.7)	8 (22.9)	5 (27.8)	
Age (years, range)	60.5 (36–74)	63 (37–78)	64.5 (41–85)	0.620
BMI (kg/m^2^, Mean ± SD)	21.50 ± 4.04	22.46 ± 3.17	21.77 ± 2.99	0.611
Underlying diseases				0.182
Yes	7 (58.3)	13 (37.1)	11 (61.1)	
No	5 (41.7)	22 (62.9)	7 (38.9)	
Chemoradiotherapy history				0.663
Yes	10 (83.3)	24 (68.6)	12 (66.7)	
No	2 (16.7)	11 (31.4)	6 (33.3)	

*BMI* body mass index, *kg* kilogram, *m* meter, *SD* standard deviation

In terms of operation time and postoperative length of hospital stay, there was no statistically significant difference between the wedge resection group and the stapler group (P > 0.05); but the operation time and postoperative length of hospital stays of the wedge resection group were shorter than those of the hand suture group (91.17 ± 15.99 min vs. 118.50 ± 28.00 min, P = 0.001; 5.58 ± 1.31 days vs. 7.50 ± 2.15 days, P = 0.007, respectively); and those of the stapler group were also shorter than the hand suture group (93.69 ± 20.26 min vs. 118.50 ± 28.00 min, P < 0.001; 4.63 ± 1.11 days vs. 7.50 ± 2.15 days, P < 0.001, respectively).

In terms of surgical consumable cost, there was no statistically significant difference between the wedge resection group and the hand suture group (2253.82 ± 794.60 yuan vs. 1954.88 ± 1005.96 yuan, P = 0.469). However, the cost in the wedge resection group was significantly lower than that in the stapler group, the difference was statistically significant (2253.82 ± 794.60 yuan vs. 8008.05 ± 1223.51 yuan, P < 0.001).

In terms of postoperative exhaustion time, the wedge resection group had an earlier recorded time than the stapler and hand suture groups, with statistically significant differences (2.08 ± 0.51 days vs. 2.49 ± 0.51 days, P = 0.029, and 2.08 ± 0.51 days vs. 2.61 ± 0.61 days, P = 0.011, respectively).

By contrast, there were no statistically significant differences in terms of intraoperative blood loss among the three groups (P = 0.822). The data of the three patient groups are shown in Table 2. In this study, no patients died due to ileostomy closure surgery. The total postoperative complication incidence was 12.31%, and the difference between the groups was not statistically significant (0, 11.43%, 22.22%, P = 0.226). The specific data of postoperative complications are shown in Table 3. Moreover, the three patient groups did not require a second operation or readmission for postoperative complications within 30 days after surgery.

**Table 2:** Data analysis in the three groups

	Wedge resection (n = 12, %)	Stapler (n = 35, %)	Hand suture (n = 18, %)	*P* value
Operation time (min)	91.17 ± 15.99	93.69 ± 20.26	118.50 ± 28.00	< 0.001
Intraoperative blood loss (ml)	15.00 ± 4.77	24.14 ± 21.54	27.50 ± 25.22	0.822
Surgical consumables cost (yuan)	2253.82 ± 794.60	8008.05 ± 1223.51	1954.88 ± 1005.96	< 0.001
Postoperative exhaustion time (days)	2.08 ± 0.51	2.49 ± 0.51	2.61 ± 0.61	0.031
Postoperative length of hospital stays (days)	5.58 ± 1.31	4.63 ± 1.11	7.50 ± 2.15	< 0.001
Postoperative complication incidence				0.226
Yes	0 (0)^a^	4 (11.4)^a^	4 (22.2)^a^	
No	12 (100)^a^	31 (88.6)^a^	14 (77.8)^a^	

*min* minute, *ml* milliliter

^a^Each subscript indicated a subset of the postoperative complication categories, and there was no significant difference between the column proportions of these categories

**Table 3:** The incidence and mortality of postoperative complications in the three groups

	Wedge resection (n = 12, %)	Stapler (n = 35, %)	Hand suture (n = 18, %)
Death	0	0	0
Anastomotic leakage	0	0	0
Anastomotic bleeding	0	0	0
Intestinal obstruction	0	0	0
Postoperative continuous vomiting	0	0	0
Postoperative abdominal pain and distension^a^	0	0	1 (5.6)
Wound infection	0	1 (2.9)	0
Postoperative fever (> 38.2 °C)^a^	0	3 (8.6)	4 (22.2)

^a^Multiple complications can occur simultaneously in one person

## Discussion

The safety and feasibility of using stapler approach and hand suture approach for ileostomy closure have been recognized by the majority of surgeons [11, 14]. Each approach has their own advantages and disadvantages, and there is no optimal choice for ileostomy closure which usually depends on the experience and willingness of clinical centers and surgeons.

In this study, there was little intraoperative blood loss in the three groups, and was no statistical difference between groups. However, both stapled and hand suture approaches required mesenteric vessel ligation and disconnection, which objectively increases the bleeding risk. By contrast, the bleeding risk of wedge resection approach is lower, and the safety is higher.

The total incidence of postoperative complications (including postoperative abdominal pain, abdominal distension, postoperative fever, and wound infection) in this study was 12.31%, which was similar to that of other experience centers [13, 17, 18]. And the 65 patients in our center did not occur postoperative ileus and other severe complications. Although there were no significant differences in the incidence among the three groups, the postoperative complication rate of the stapler group or the hand suture group was higher than that of the wedge resection group (11.43% and 22.22% vs. 0%, respectively). All patients with postoperative complications become better after active treatment.

Adequate blood supply is the most important factor for establishing intestinal anastomosis [19]. It is well known that the blood transport of the small intestine comes from the blood vessels in the mesentery which finally enter the wall of the small intestine from the mesangial margin as straight arteries perpendicular to the longitudinal axis of the intestinal canal. As such, compared to stapler and hand suture approaches, the wedge resection plus transverse suture without mesentery detached approach does not require mesenteric vessel ligation and disconnection, which maximizes the anastomotic blood supply in the most physiological manner while avoiding anastomotic leakage caused by intestinal ischemia [20]. However, the stapler anastomosis or hand suture end-to-end anastomosis requires resecting the intestine and part of the stoma mesentery, resulting in a certain distance between anastomotic stoma and the mesenteric margin. Theoretically, this distance should not be greater than 1.0 cm; otherwise, it would be extremely easy to occur anastomotic leakage caused by ischemia of the anastomotic stoma [20]. For hand-sewn end-to-end anastomosis, the diameter of the intestine segment at the distal end of the stoma can be significantly reduced owing to its prolonged exclusion; however, the diameter of the two ends is inconsistent, which can also increase the risk of anastomotic leakage or stenosis.

Studies including that of Löffler et al. have observed that stapler anastomosis taked less time than hand suture anastomosis, which made performing the procedure more conveniently [10, 11]. Although the intestine needs to be resected in stapler anastomosis, the resection of the ostomy intestine and the closure of the common opening are conveniently and quickly completed at one time, which can shorten the time of intestine anastomosis. Meanwhile, the wedge resection without mesentery detached approach did not require mesenteric vessel ligation and intestine resection, it only required trimming of the stoma edge with manually transverse suturing later. And only part of the intestine needed to be sutured, so there was no difference in the operation time between the stapler group and wedge resection group. Compared with the wedge resection approach or the stapler approach, the hand suture approach required mesenteric vessel ligation, intestine resection and full-peripheral intestinal suture, the operation time required was the longest, which is consistent with the data of this study.

The gastrointestinal motility of the three groups recovered to normal after surgery, and the patients’ postoperative exhaust time was within 1–4 days; however, the patients who underwent wedge resection plus transverse suture without mesentery detached approach had an earlier postoperative exhaustion time. This may be because the intestine was not disconnected by this approach. Moreover, the operation was completed in the most suitable way for intestinal physiology, reducing nerve plexus damage in the intestinal wall [21]. After the operation, the intestinal wall of the stoma could still be quickly adjusted using the nerves to promote the peristalsis of the smooth muscle of the small intestine [22].

With the implementation of the DRG payment method, the medical insurance payer no longer pays according to the patient’s actual hospital expenditure, but he now pays according to the related groups, such as type, severity, treatment, and other conditions, of the patient’s disease [23, 24]. Under the premise of ensuring patient quality treatment and to save medical costs and reduce economic burden, hospitals need to actively reduce and control treatment costs. In this study, the wedge resection and hand suture groups only needed to apply surgical sutures to complete intestinal anastomosis with low cost, while the stapler group required 2 pieces of 80 mm anastomosis nails, making the anastomotic cost higher. Moreover, the wedge resection group only needed to suture part of the intestine transversely without involving the mesenteric intestinal wall and blood vessels, among others, while the hand suture anastomosis group required a full-peripheral intestinal anastomosis, needing more sutures in a wider range. Therefore, compared with stapled anastomosis and hand suture anastomosis, wedge resection plus transverse suture was more economical and cost-effective.

In previous years, wedge resection was rarely used in the closure of ileostomy, which may have been due to issue about surgical factors causing anastomotic stenosis. We believe that after trimming the edges of the stoma and removing the adhesive skin and tissues, the possibility of postoperative anastomotic stenosis becomes very low when using the transverse suture method following the traditional surgical principle of “longitudinal resection and transverse suture” [25]. Additionally, the anastomotic ring can intraoperatively accommodate a transverse finger for all patients after the intestinal repair is performed, given that no case of postoperative anastomotic stenosis has been attributed to this. In the past, considering the serious adhesion between the stoma and the abdominal wall, it was difficult to separate the normal intestinal tube without damage, and the wedge resection plus transverse suture method was not used. Despite this, for a skilled attending physician or a more senior doctor, it was not a problem to avoid intestinal injury through delicate surgical operations. Therefore, wedge resection plus transverse suture, as a surgical method for ileostomy reversal, is safe and easy to perform, has sufficient advantages for anastomotic blood supply, and it is a surgical method worth exploring.

Despite these findings, the study has certain limitations because the sample size was small and the evidence strength was not high, which may even have promoted selection bias. In the future, there is a need for randomized controlled trials with expanded sample sizes and long-term follow-ups to verify the feasibility and advantages of this surgical method.

In conclusion, wedge resection plus transverse suture without mesentery detached approach is easy to operate, has reliable blood supply, does not increase postoperative complication incidence, and allows quick recovery of the intestinal motility after surgery. Moreover, it is safe and feasible for closure of loop ileostomy in selected patients, and the cost of consumables required for surgery is small, making it particularly suitable for the DRG payment method for medical insurance. Thus, this is a recommended approach for closure of loop ileostomy in selected patients.

## Data Availability

The data that support the findings of this study are available from the corresponding author upon reasonable request.
